# Preparation and Application of pH-Sensitive Film Containing Anthocyanins Extracted from *Lycium ruthenicum* Murr.

**DOI:** 10.3390/ma16103828

**Published:** 2023-05-18

**Authors:** Yucong Zhao, Le Gao, Jing Wang, Ziyan Xue, Mengyao Zhang, Xueli Ma, Guohua Wang, Shenghua Lv

**Affiliations:** College of Bioresources Chemical and Materials Engineering, Shaanxi University of Science and Technology, Xi’an 710021, Chinamxl01051128@163.com (X.M.);

**Keywords:** *Artemisia sphaerocephala* Krasch. gum, soybean protein isolate, *Lycium ruthenicum* Murr., anthocyanin extract, pH-sensitive film

## Abstract

A new pH-sensitive film was developed using *Artemisia sphaerocephala* Krasch. gum (ASKG), soybean protein isolate (SPI), and natural anthocyanin extracted from *Lycium ruthenicum* Murr. The film was prepared by adsorbing anthocyanins dissolved in an acidified alcohol solution on a solid matrix. ASKG and SPI were used as the solid matrix for the immobilization of the *Lycium ruthenicum* Murr. anthocyanin extract, which was absorbed into the film as a natural dye using the facile-dip method. Regarding the mechanical properties of the pH-sensitive film, the tensile strength (TS) values increased approximately 2–5-fold, but the elongation at break (EB) values decreased significantly by about 60% to 95%. With the increase in anthocyanin concentration, the oxygen permeability (OP) values first decreased by about 85%, and then increased by about 364%. The water vapor permeability (WVP) values increased by about 63%, and then decreased by about 20%. Colorimetric analysis of the films revealed variations in color at different pH values (pH 2.0–10.0). Fourier-transform infrared (FT-IR) spectra and XRD patterns indicated compatibility among ASKG, SPI, and anthocyanin extracts. In addition, an application test was conducted to establish a correlation between film color change and carp meat spoilage. At storage temperatures of 25 °C and 4 °C, when the meat was totally spoiled, the TVB-N values reached 99.80 ± 2.53 mg/100 g and 58.75 ± 1.49 mg/100 g, and the film’s color changed from red to light brown and from red to yellowish green, respectively. Therefore, this pH-sensitive film could be used as an indicator to monitor the freshness of meat during storage.

## 1. Introduction

Visual pH labels, an essential component of intelligent packaging systems, are a new concept that can provide consumers with real-time information on the safety and quality of food products [1,2]. Food spoilage can cause metabolization by microorganisms and enzymes, which produces carbon dioxide, organic acids, amines, ammonia, and other substances. This can significantly increase the total volatile basic nitrogen (TVB-N) and pH values [3,4,5]. Thus, detecting changes in pH provides a reference for consumers to quickly identify the freshness of food [6,7]. Due to their limited size, strong sensitivity, low cost, and high convenience, visual pH labels have been widely used for rapid food quality detection [8]. Visual pH labels are usually prepared from a pH-sensitive dye and a solid matrix used to immobilize the pH dye. The solid matrix is mainly derived from natural and biodegradable polymers, including chitosan, pectin, agar, and starch, which are converted into flexible and transparent thin-film thermoplastic materials under the action of plasticizers [9,10,11,12,13,14,15,16,17,18,19]. Unlike traditional chemical dyes, such as bromophenol blue and chlorophenol red, which are potentially harmful to human health, natural dyes that are low in toxicity and biodegradable have been extensively studied for use in food packaging [20,21]. Anthocyanins, which are secondary metabolites of fruits and vegetables, have a sensitive color response to a wide range of pH values [22,23]. As a result, anthocyanin dyes are the most suitable materials for the preparation of pH-sensitive films [24]. Minor modifications in the chemical structure of anthocyanins derived from different plants can lead to significant differences in the properties of the color change. Thus, the most important experimental effort is to select the optimal source of anthocyanins. At present, research mainly focuses on the performance and application of pH indicator films prepared from purple potato extracts (PSPE), grape extracts, purple cabbage extracts, black bean extracts, etc. [25,26,27,28,29,30].

*Artemisia sphaerocephala* Krasch. gum (ASKG), extracted from *Artemisia sphaerocephala* Krasch. (ASK) seeds, is a kind of macromolecular heteropolysaccharide with branched chains. Its molecular structural components are L-arabinose, D-xylose, D-lysose, D-mannose, D-glucose, and D-galactose. Due to its high viscosity, superior moisture retention, good dispersibility, and chemical and thermal stability, ASKG is an excellent packaging substrate for the preparation of edible films [31,32]. ASKG-based edible films have selective permeability to carbon dioxide and oxygen, but have relatively poor elongation at break, a shortcoming that largely limits their food packaging applications. For the best results, different biopolymers, such as polysaccharides, lipids, or proteins, are mixed to incorporate the main features and qualities of each. Proteins consist of amino-acid residues linked by peptide bonds, and they are very compatible with polysaccharides due to the interplay of hydrogen bonds between the two types of molecules [33,34]. Soy protein isolate (SPI) is extracted from soybeans. There are a large number of hydrogen and disulfide bonds within the SPI molecule. The film formed by soy protein isolation is flexible, smooth, and flat [35]. ASKG and SPI were, therefore, mixed to form an edible film with favorable properties through intermolecular hydrogen bond interactions. ASKG and SPI were used as the substrates, glycerol was added as the plasticizer, and the films were formed using the casting method.

In this study, the solid matrix was an edible composite film prepared from ASKG and SPI, which had the characteristics of strong elongation at break (EB) and excellent flexibility. The anthocyanins used as pH-sensitive dyes were extracted from *Lycium ruthenicum* Murr. Instead of the current commonly used blend casting method, a facile dip-loading technique was employed by immersing the composite film in an acid–alcohol solution of anthocyanins. The pH indicator was formed by spontaneous association of anthocyanins with the components of the solid matrix. In addition, the performance of the indicator film was evaluated by measuring its mechanical and barrier properties, the color variability at different values of pH, and the chroma colorimetry of the NH_3_ response. Furthermore, the colorimetric films were characterized by Fourier-transform infrared spectroscopy (FT-IR), scanning electron microscopy (SEM), and X-ray diffraction.

## 2. Materials and Methods

### 2.1. Materials

*Artemisia sphaerocephala* Krasch. gum (ASKG) was purchased from Xinxing Biotechnology (Inner Mongolia, China). Soybean protein isolate (SPI) was purchased from Yuwang Group (Shandong, China). Glycerol (analytical reagent) and ammonia aqueous (guaranteed reagent) were purchased from Sinopharm Chemical Reagent Co., Ltd. (Shanghai, China).

### 2.2. Extraction of Anthocyanins

*Lycium ruthenicum* Murr. anthocyanin (LRMA) was extracted by following the standard extraction procedure with some modifications [36,37]. Briefly, 50 g of dried *Lycium ruthenicum* Murr. was macerated with 250 mL of distilled water at 50 °C for 30 min. The pulp obtained by homogenization was added to 250 mL of 70% (*v*/*v*) ethanol at 50 °C for 30 min with stirring. After this period, the extracted solution was filtered with Whatman filter paper, and the filter residue was centrifuged at 2.7 × 10^3^× *g* for 20 min to collect the supernatant. The filtered liquid was mixed with the supernatant, and the resulting solution was concentrated using a rotating evaporator. The concentrated extract solution was kept at −60 °C for about 2 h, and then freeze-dried.

### 2.3. Preparation of Films

On the basis of preliminary experimental results, the ASKG solution was obtained by dissolving 0.6 g of ASKG powders in 100 mL of distilled water at 60 °C for 2 h under magnetic stirring. Meanwhile, the SPI solution was prepared by dispersing 5 g of SPI powders in 100 mL of distilled water at 80 °C for 30 min under magnetic stirring. Then, the film-forming solution was obtained by mixing the ASKG solution with the SPI solution at a volume ratio of 1:1, and adding 40% (*w*/*w*, on a dry basis of ASKG and SPI) glycerol. After stirring for 10 min, the film-forming solution was cast onto an acrylic plate (20 cm × 20 cm × 1.5 cm) and left to dry at ambient temperature for 12 h until the surface was solidified; then, the films were submitted to hot air drying at 50 °C for 12 h. Prior to determining the mechanical and barrier performances, the composite films were stored at a temperature of 25 °C and a relative humidity (RH) of 75% for 24 h, and the moisture content was 0.073 ± 0.003 (g/g).

The pH-sensitive film was prepared by immersing the composite film in an acidified ethanol solution (1.46 mL of hydrochloric acid in 100 mL of 50% ethanol) with anthocyanin extracts of *Lycium ruthenicum* Murr. at concentrations of 1%, 2%, 3%, and 4% (g/mL) for 30 min. After removal from the acidified ethanol solution, the surface of the pH-sensitive films was rinsed with 50% (*v*/*v*) acidified ethanol and air-dried; then, they were stored at a temperature of 25 °C and an RH of 75% for 24 h, with a moisture content of 0.030 ± 0.002, 0.015 ± 0.001, 0.017 ± 0.004, and 0.040 ± 0.005 g/g, respectively. Depending on the concentration of anthocyanin solution, the pH-sensitive films were described as LRMA-1, LRMA-2, LRMA-3, and LRMA-4. In the NH_3_-sensing test, the LRMA-3 film was selected and equilibrated for 24 h at an RH of 33%, 75%, and 86%, with a moisture content of 0.010 ± 0.001, 0.017 ± 0.004, and 0.023 ± 0.005 g/g, respectively.

### 2.4. Mechanical Properties

The thickness of each film was measured using a CH-1-S millimeter thickness gauge (Shanghai Liuling Instrument Factory, Shanghai, China), and the thickness values of the films were taken as the average of five random points [38]. The films were cut into rectangular strips of 150 mm × 15 mm (Chinese National Standard: GB/T 1040.1-2018, GB/T 1040.3-2006). After conditioning at 25 °C and RH of 75% for 24 h, the tests of mechanical properties (tensile strength and elongation breaks) were carried out at a speed of 10 mm/min with a tensile testing machine (XLW-PC, PARAM, Jinan, China) [39].

### 2.5. Barrier Properties

The water vapor permeability (WVP) of a film was tested according to the Chinese National Standard (GB/T 26253-2010). Briefly, the sample was sealed in a cup containing distilled water, and then a test cup containing dry calcium chloride was placed over the films. The mass of moisture was weighed at 38 °C and an RH of 90%.

The oxygen permeability (OP) of the films was tested in accordance with the Chinese National Standard (GB/T 28765-2012). First, a thin film was cut into a circular sample of 150 mm diameter and sealed on the instrument with hot melt glue. Then, the test mode was set to high resistance with the following parameters: temperature 25 °C, relative humidity 0%, flow rate of oxygen 10 mL/min, purge time 30 min.

### 2.6. Determination of Total Volatile Basic Nitrogen (TVB-N)

The TVB-N values were determined using the Chinese National Standard method (GB5009.228-2016). After the skin and bones were removed, 10 g of fish meat was weighed and placed in a digestive tube, to which 75 mL of deionized water was added. The sample was digested using a K9840 Automatic Kieldahl apparatus (Hanon Advanced Technology Group Co., Ltd., Jinan, China) and titrated with 0.1 mol/L hydrochloric acid solution to an endpoint of pH 4.65. The amount of TVB-N was determined using Equation (1).

(1) $$X=\frac{\left(V_{1}-V_{2}\right)\times C\times 14}{m}\times 100$$

where *X* is the weight (mg) of TVB-N per 100 g of fish samples, *V*_1_ is the volume (mL) of standard hydrochloric acid solution consumed by a sample titration, *V*_2_ is the volume (mL) of standard hydrochloric acid solution consumed by a control titration, *C* is the molar concentration (mol/L) of standard hydrochloric acid solution, 14 is the mass of nitrogen equivalent to 1.0 mL of standard hydrochloric acid solution, *m* is the weight (mg) of the sample, and 100 is a unit conversion coefficient.

### 2.7. Colorimetric Analysis

The color spectrum was determined using a CM-5 colorimeter (Konica Minolta Holdings, Inc., Tokyo, Japan), and expressed in terms of L* (lightness), a* (red to green), and b* (yellow to blue) values to assess the color changes at pH values of 2.0–10.0. According to the method of CIE *Lab*, the total color difference (Δ*E*) was calculated using the following Equation (2) [40]:

(2) $$\Delta E=\sqrt{{\left({\Delta L}^{*}\right)}^{2}+{\left({\Delta a}^{*}\right)}^{2}+{\left({\Delta b}^{*}\right)}^{2}},$$

where Δ*E* is the total color difference, L*, a*, and b* are color values of the LRMA in each pH buffer solution, and *L*_0_*, *a*_0_*, and *b*_0_* are the initial color values.

To detect the color spectrum of pH-sensitive films exposed to buffer solutions of different pH values, the films were cut into 30 mm × 30 mm square-shaped plates which were then immersed in 5 mL of buffer solution ranging from pH 2.0 to pH 10.0 for 5 min. The film color was measured after removing the buffer solutions and blotting with filter paper [41].

In the NH_3_-sensing test, square strips (30 mm × 30 mm) of the films were placed in containers with relative humidities of 33%, 75%, and 86% at room temperature for 12 h. Then, 5 mL of 25% (*w*/*w*) ammonia aqueous was injected into the beaker, and the film color change was recorded at set intervals by means of a colorimeter.

All measurements were taken at three random locations on both sides of each sample. Tests were carried out in triplicate.

### 2.8. Fourier Transform Infrared (FT-IR) Spectra

The FT-IR spectra of samples were scanned using a spectrometer (Bruker-VERTEX 70, Bruker Optic GmbH, Karlsruhe, Germany) under an attenuated total reflectance (ATR) mode. The wavenumbers were measured with 16 scans from 400 to 4000 cm^−1^ with a resolution of 4 cm^−1^ [42].

### 2.9. Scanning Electron Microscopy (SEM)

Films were fractured in liquid nitrogen before the cross-sectional morphology was observed. The films were then coated with gold, and the morphologies of the surface and the cross-section were observed using a scanning electron microscope (SEM) (Jeol, JSM-6610LV, Tokyo, Japan) under an accelerating voltage of 10 kV at 5000× and 3000× magnification, respectively.

### 2.10. XRD Measurement

An XRD (D8, Bruker AXS, Karlsruhe, Germany) pattern with CuKα radiation was used to characterize the information about the crystal structure of films. The diffraction angle (2θ) of samples was scanned in the range of 5–50° with a scanning rate of 4°/min.

### 2.11. Statistics Analysis

Experimental data were processed using SPSS (v26.0, SPSS Inc., Chicago, IL, USA). Duncan’s multiple-range tests (*p* < 0.05) were used to compare the differences among data. All data were expressed as mean ± standard deviation (SD).

## 3. Results and Discussion

### 3.1. UV–Vis Spectra of Lycium ruthenicum Murr. Anthocyanin (LRMA) Solution

**Figure 1:**
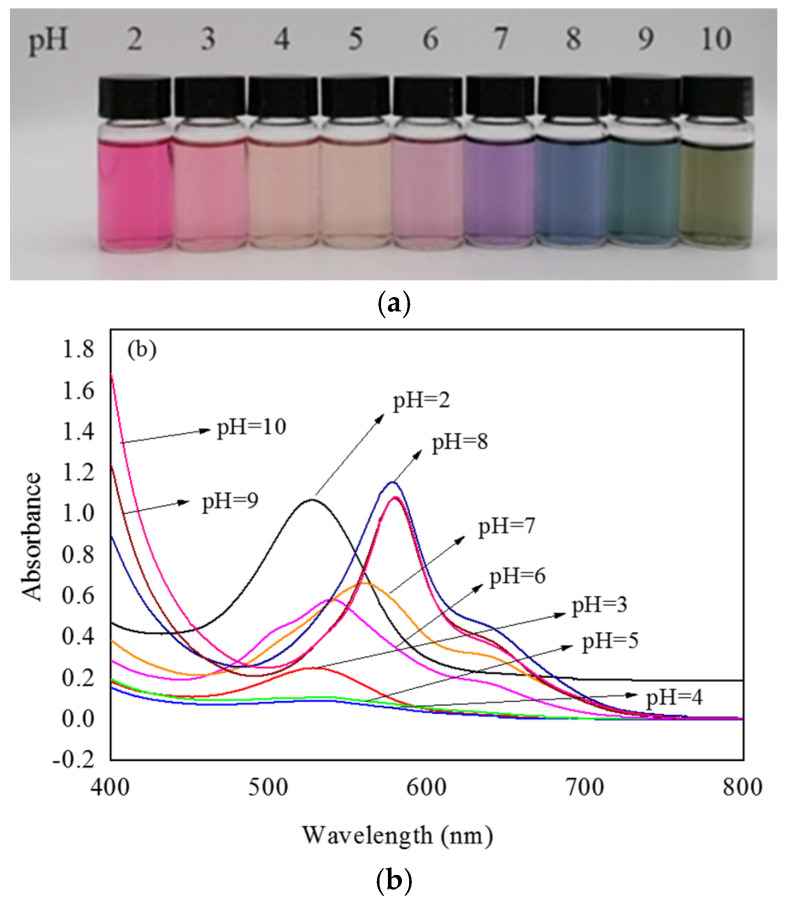
UV-Vis spectra of *Lycium ruthenicum* Murr. anthocyanin solution: (**a**) color variations; (**b**) UV-Vis spectra of *Lycium ruthenicum* Murr. extract (LRMA) solutions in pH range of 2.0–11.0.

Anthocyanins derived from diverse natural materials may have slightly different hydroxyl and methoxy groups in their chemical structure, and this small difference can have a large impact on color change behavior. The shift in the maximum absorption peak of anthocyanin solution with changes in pH is known as the bathochromic shift. Color variations in solutions of *Lycium ruthenicum* Murr. extract were tested, and the feasibility of the extract as a pH indicator dye was verified. Figure 1a shows the colors exhibited by the LRMA solution with pH increasing from 2.0 to 10.0. The color of the LRMA solution was dark pink at pH 2, pale pink at pH 3, reddish brown at pH 4–5, mauve to purple at pH 6–7, dark blue to light blue at pH 8–9, and yellow-green at pH 10. This is because the structure of anthocyanins changes successively from a flavylium cation, a quinoid base, and a carbinol pseudo-base to a chalcone [43,44]. Moreover, Figure 1b shows that the maximum absorption peak of the LRM shifts from 527 nm at pH 2.0 to 578 nm at pH 10.0. Due to their sensitivity to changes in pH, which gives rise to variations in coloration from red to green, anthocyanin-containing ASKG/SPI films have potential applications in monitoring food quality.

### 3.2. Mechanical and Barrier Properties

Table 1 shows the thickness and mechanical and barrier properties of the different films immersed in solutions of anthocyanin at different concentrations. As anthocyanins were absorbed, the thickness of the pH-sensitive film increased significantly from 0.056 ± 0.001 mm to a maximum of 0.118 ± 0.001 mm (*p* < 0.05). It is possible that the addition of anthocyanins reduced the interaction between the molecules formed by *Artemisia sphaerocephala* Krasch. gum (ASKG) and soybean protein isolate (SPI), destroying the reticular spatial structure of solid matrix [45,46]. The tensile strength (TS) value of the prepared pH-sensitive film increased considerably by a factor of approximately 2–5, while the elongation at break (EB) value decreased sharply by about 60–95%. This may have been caused by the gradual adsorption of anthocyanin onto the solid matrix molecules when the composite film was immersed in anthocyanin solution. Under the influence of intermolecular hydrogen bonds, the rigidity of the film structure was enhanced, and the number of molecules that could flow was reduced. In addition, the acid–alcohol solution weakened the protein–water interaction and strengthened the SPI intermolecular bonds. This helped the film to form a relatively dense network structure.

As the anthocyanin concentration increased, the oxygen permeability (OP) of pH-sensitive films presented an initial decrease followed by an increase, whereas the water vapor permeability (WVP) showed an increase first and then a decrease. A possible reason for this is that when the film was immersed in a low-concentration anthocyanin solution, the anthocyanin molecules were inserted into the two-sided surface of the film via hydrogen bonds, and the hydrogen-bond interactions between molecules enhanced the density of the film surface. However, in high-concentration solutions, the hydrophobic interaction between the benzene rings of anthocyanins and the aliphatic and aromatic amino acids of proteins increased, which reduced the permeability to water molecules but promoted the dissociation of oxygen and increased its transport efficiency [47,48].

### 3.3. Color Response Efficiency and Colorimetric Analysis of pH-Sensitive Films

#### 3.3.1. pH Response in Different Buffer Solutions

**Figure 2:**
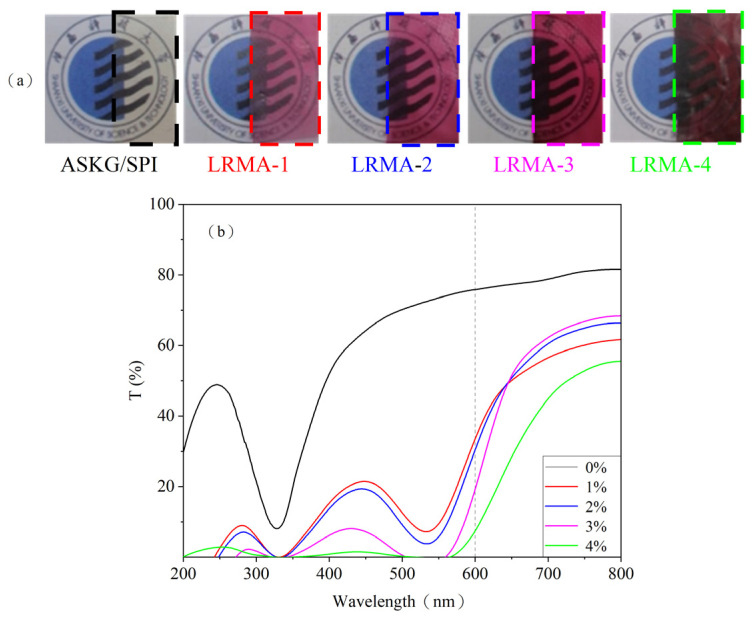
(**a**) Effect on light transparency of ASKG/SPI film and pH-sensitive films; (**b**) light transmission curves of ASKG/SPI film and pH-sensitive films.

Light transmittance is one of the important properties of food packaging materials, which can directly impact on consumer acceptability [49,50,51]. Figure 2 shows photos of films with different anthocyanin solution contents and light transmission curves. As can be seen in Figure 2a, the ASKG/SPI film was highly transparent; the transparency of the anthocyanin-containing films was gradually reduced with the increase in the anthocyanin solution content. When the concentration of anthocyanin solution was below 3%, the images of the school badge covered by the pH-sensitive films were still clearly visible. The regular light transmittance curves of the films are shown in Figure 2b, where the results for films with different anthocyanin contents show significant differences. In the wavelength range of UV light, the transmittance of ASKG/SPI films reached 39% at 260 nm, and the transmittance of anthocyanin-containing ASKG/SPI films decreased from 18% to approximately zero at 270 nm. This shows a strong UV light-shielding effect when a certain amount of LRMA was adsorbed onto the ASKG/SPI film. Similarly, in the wavelength range of visible light, the transmittance of the ASKG/SPI film reached 77% at 600 nm, but the transmittance of the anthocyanin-containing film decreased from 36% to 8% at 600 nm with an increase in LRMA solution content from 1% to 4%. One possible reason is that LRMA disrupts the ordered arrangement of molecular chains formed by ASKG and SPI, leading to increased scattering and refraction of light [52,53]. These results suggest that anthocyanin-containing films exhibit a shielding effect against UV light and have potential applications in reducing the food spoilage that it can cause.

#### 3.3.2. Visual Color Response of pH-Sensitive Film

Table 2 shows the colorimetry parameters obtained by immersing the film in different buffer solutions. It reveals that, as the concentration of anthocyanin solution increased, the value of L* gradually decreased and the color of the film deepened under the same pH response. When comparing colorimetric parameters (L*, a*, and b*) of the films prepared by four concentration gradients, the LRMA-3 film had the best visualization performance. Values of parameter a* show that red decreased first and then increased with pH, while yellow showed the opposite trend; values of parameter b* show that blue decreased first and then increased with pH, while yellow showed the opposite trend. In addition, the film became dark pink at pH 2.0 and light pink at pH 3.0. When it was soaked in higher-pH buffer solutions (pH 4.0–6.0), the color of the film deepened from a light purplish brown to an intense purplish brown. The color changed from purple to blue-purple in a buffer solution of pH 7.0–10.0.

#### 3.3.3. Color Response in NH_3_ Atmosphere

**Figure 3:**
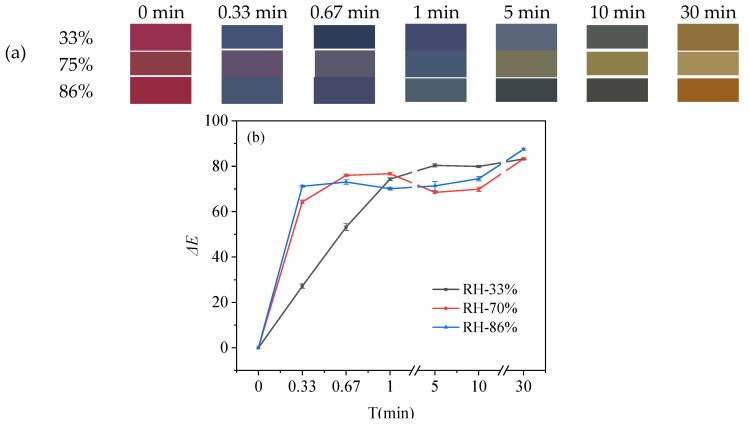
(**a**) Color variations for different time periods at 33%, 75%, and 86% relative humidity; (**b**) Δ*E* values for different time periods at 33%, 75%, and 86% relative humidity.

Since volatile nitrogen compounds are released during the decomposition of protein-rich foods, NH_3_-response tests were performed on the indicator films to assess the colorimetric response of these compounds. On the basis of the above experimental results, a film of LRMA-3 was selected to investigate the sensitivity of ammonia vapor at different relative humidity levels (33%, 75%, and 86%). The color of the film changed from reddish-pink to lake blue, purple, dark blue, blue-gray, pale blue-gray, cyan-blue, yellow-green, and brown within 30 min of exposure to ammonia vapor, as shown in Figure 3a [54]. When the relative humidity increased from 33% to 88%, there was a greater variation in Δ*E* values, and it became easier to observe the color change with the naked eye [55]. Figure 3b shows that the Δ*E* values were 27.11 ± 1.01, 64.30 ± 0.82, and 71.14 ± 0.40 within 20 s under the relative humidity values of 33%, 75%, and 86%, respectively. The Δ*E* values reported from the casting method were 7.92, 36.48, and 35.77 within 10 min under relative humidity values of 33%, 75%, and 90%, respectively [53]. On comparing the results of the two methods, the pH-sensitive film prepared by a facile-dip method showed a faster NH_3_-response speed. These results were due to the alkaline conditions resulting from the presence of ammonium ions on the film surface, as well as the higher relative humidity promoting hydration–hydrolysis of ammonia vapor and formation of hydroxide anions.

### 3.4. XRD Patterns of ASKG/SPI and LRMA-Containing ASKG/SPI-Blended Film

**Figure 4:**
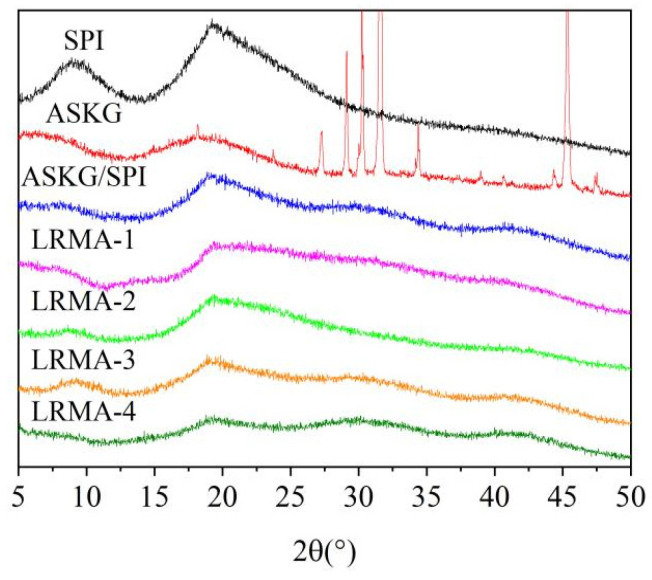
XRD patterns of SPI, ASKG, ASKG/SPI films, and pH-sensitive films (LRMA-1, LRMA-2, LRMA-3, and LRMA-4).

The XRD patterns of SPI, ASKG, ASKG/SPI films, and LRMA-containing films are shown in Figure 4. The XRD pattern of SPI showed a wide diffraction peak at about 2θ 8.5°, and a sharp and wide diffraction peak at 2θ 19°. The two characteristic peaks corresponded to the lateral α-helix packing and β-sheet structures of the secondary structure, respectively, which were previously confirmed in soy protein, zein, and locust protein [56,57].

ASKG powder mainly exists in an amorphous α structure, and its XRD pattern shows a broad dispersion peak at 2θ 20° [58]. The XRD pattern of the ASKG/SPI film showed a decrease in diffraction peak intensities at 2θ 8.5° and 2θ 19°, caused by hydrogen-bond interactions between ASKG and SPI. No significant effect was observed on the XRD diffraction peak position after immersion in the anthocyanin solution, with only a decrease in the intensity of the diffraction peak. An increase in LRMA content caused the diffraction peaks at 2θ 8.5° and 2θ 19° to decline; this was especially apparent when LRMA solution content reached 4%, at which point the peak at 2θ 8.5° disappeared entirely, indicating that the intramolecular connection and the original ordered crystal structure were destroyed to some extent [59]. Thus, the incorporation of anthocyanins disrupted the intermolecular and intramolecular hydrogen bonds of ASKG/SPI and reduced crystallinity, the destruction of which was closely related to the concentration of anthocyanin solution [37,60,61].

### 3.5. FT-IR Spectra of pH-Sensitive Film

**Figure 5:**
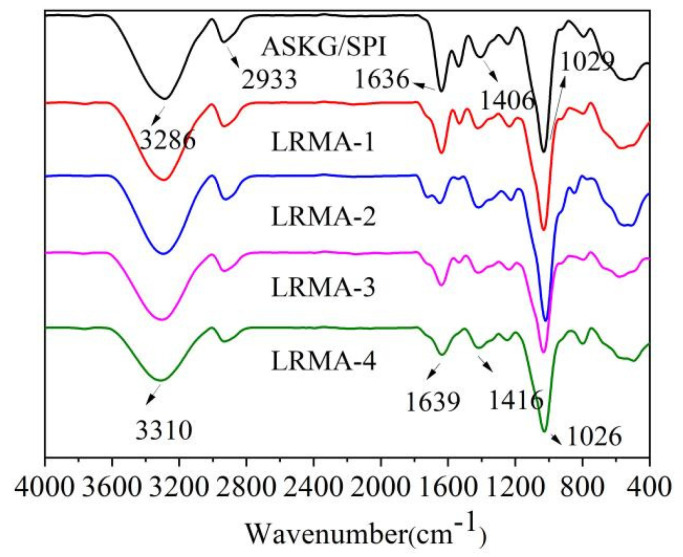
FT-IR spectra of ASKG/SPI and pH-sensitive films (LRMA-1, LRMA-2, LRMA-3, and LRMA-4).

The FT-IR spectra of the pH-sensitive film immersed in different concentrations of LRMA are shown in Figure 5. The characteristic bands of the ASKG/SPI-blended film (control) were 3286 cm^−1^ due to O–H stretching and 2933 cm^−1^ due to C–H stretching. The characteristic bands of the LRMA-containing ASKG/SPI-blended film were 3310 cm^−1^ due to O–H stretching and 2933 cm^−1^ due to C–H stretching [62,63]. After the addition of anthocyanins, the stretching vibrations of the hydroxyl group caused a hypochromic shift. This suggests that the molecular interactions between the anthocyanins and the solid matrix of the film led to a shift of the C–H stretching peak toward higher wavenumbers [64]. In addition, the bands at 1639 cm^−1^ and 1636 cm^−1^ were assigned to C=C vibration, those at 1416 cm^−1^ and 1406 cm^−1^ were assigned to C–N stretching vibration, and those at 1029 cm^−1^ and 1026 cm^−1^ were assigned to C–O–C stretching vibration [65]. Moreover, the absorbance of C–H stretching, C=O stretching, and C–O–C stretching decreased significantly with increasing anthocyanin solution concentration, attributed to the formation of new bonds between the molecules [66].

### 3.6. Scanning Electron Microscopy (SEM)

**Figure 6:**
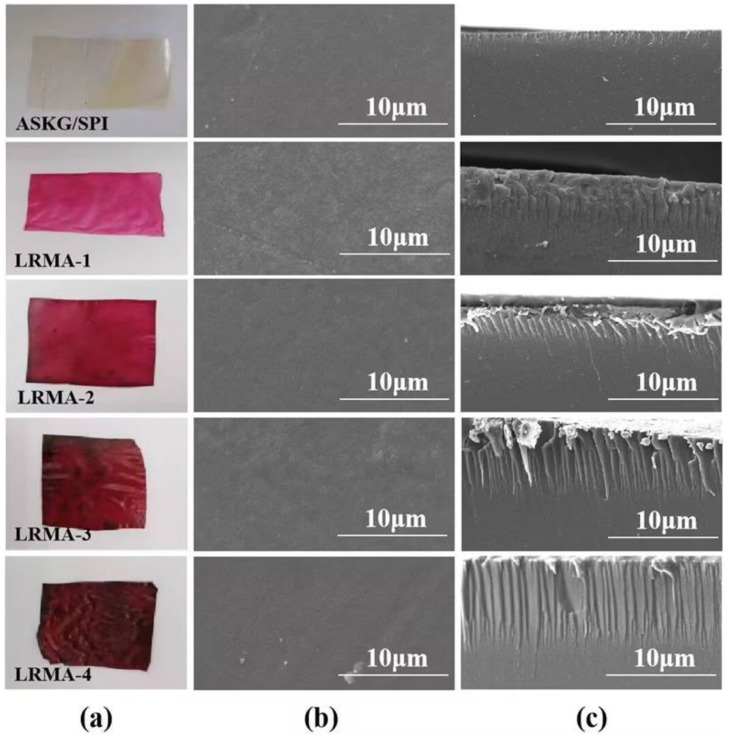
SEM images of ASKG/SPI film and pH-sensitive films (LRMA-1, LRMA-2, LRMA-3, and LRMA-4): (**a**) samples; (**b**) surface SEM images; (**c**) cross-sectional SEM images.

The morphologies of the surface and cross-sections of the ASKG/SPI film and the LRMA-containing pH-sensitive films are shown in Figure 6. The ASKG/SPI film showed a smooth and homogeneous surface and cross-section morphology. In contrast, as the LRMA content increased, the surface of the LRMA-containing film became rougher and less uniform, and a few small granules were also observed. At the same time, the cross-sectional profile showed tiny cracks on the outer surface of the LRMA-containing film, which gradually lengthened. A possible reason for this is that, at low concentrations of LMRA solution, the anthocyanin molecules interacted only with the surface molecules of the solid matrix [67,68]. Upon increasing the strength of the solution, the number of anthocyanin molecules aggregating on the surface of the film increased, and the anthocyanin molecules entered the interior of the film via hydrogen bonding, breaking the original compact spatial structure between ASKG and SPI [46,53].

### 3.7. Correlation Study between Meat Spoilage and Colorimetric Change of pH-Sensitive Film

Colorimetric change is an important aspect of intelligent packaging applications, where pH-sensitive films are used to monitor meat quality during storage and distribution. Due to the action of microorganisms and enzymes, meat products gradually undergo spoilage which leads to the formation of volatile nitrogenous compounds, such as ammonia and amines [69,70]. Since the total volatile basic nitrogen (TVB-N) and pH values of spoiled meat significantly increase, it is possible to use TVB-N and pH values as reference indices for meat spoilage [71,72].

**Figure 7:**
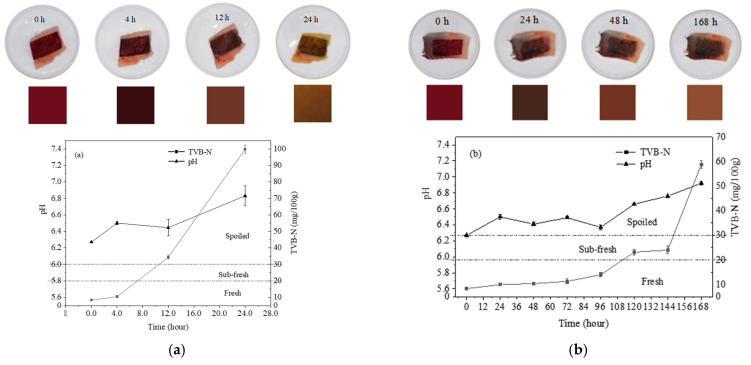
Color changes of the pH-sensitive films; pH and TVB-N values of grass carp meat samples: (**a**) Storage temperature at 25 °C; (**b**) Storage temperature at 4 °C.

A test was carried out to correlate colorimetric changes of pH-sensitive films with changes in TVB-N and pH in grass carp meat under storage temperatures of 25 °C and 4 °C, respectively [29,73]. Figure 7 depicts the correlation between the colorimetric changes of the pH-sensitive film and the pH changes of the grass carp meat. The initial (day 0) color of the pH-sensitive film was red, the TVB-N value of the fresh fish meat was 8.33 ± 0.51 mg/100 g, and the pH value was 6.27 ± 0.01. Figure 7a shows that, after 4 h storage at room temperature (25 °C), the TVB-N value of fish meat was 10.41 ± 0.38 mg/100 g, the pH was 6.50 ± 0.02, and the color of the film had darkened; by this time, the fish was still in a fresh state. When stored for 12 h, the fish had a TVB-N value of 34.36 ± 1.20 mg/100 g and a pH of 6.45 ± 0.10, and the film color changed from dark red to brown; this indicated that the fish was decaying when the TVB-N value was greater than 30 mg/100 g. After 24 h of storage, the fish was entirely spoiled. The TVB-N and pH values increased to 99.80 ± 2.53 mg/100 g and 6.83 ± 0.12, respectively. The color of the film turned to light brown. Because storage at low temperature can extend shelf-life, an additional experiment was conducted to investigate spoilage at 4 °C. The results are shown in Figure 7b. The fish remained fresh for 96 h, after which it slowly began to decay in storage from about 100 h to 144 h. The full spoilage point was finally reached after 168 h of storage, at which point the TVB-N value was 58.75 ± 1.29 mg/100 g and the pH was 6.92 ± 0.02. The color of the film changed sequentially from red to deep red, reddish-brown, brown, and yellow-green. Thus, this colorimetric pH-sensitive film can be used as a reliable method to monitor the spoilage of meat products with the naked eye.

## 4. Conclusions

In this study, two biodegradable materials, soybean protein isolate and *Artemisia sphaerocephala* Krasch. gum, were used in the development of composite films. Instead of the usual casting method, the pH-sensitive film was prepared using a facile-dip method in which the composite film was immersed in an acidified ethanol solution containing anthocyanin extracts from *Lycium ruthenicum* Murr. The use of 50% (*v*/*v*) acidified ethanol solution could also prevent the disintegration of the composite film in aqueous solution, as well as facilitate the adsorption of anthocyanins into the solid matrix. The immobilization of anthocyanins led to an increase in the TS values and a decrease in the EB values of the films. The XRD results showed that the electrostatic interactions among LRMA, ASKG, and SPI affected the crystal structure of the films. The SEM analysis indicated that tiny cracks appeared in the cross-section of the film, which gradually lengthened as the anthocyanin solution concentration increased. Furthermore, the indicator film showed high sensitivity and distinct color variation. In NH_3_ response tests, the Δ*E* values were 27.11 ± 1.01, 64.30 ± 0.82, and 71.14 ± 0.40 at relative humidity levels of 33%, 75%, and 86%, respectively, within 20 s, and colors changed from red to blue-gray. The NH_3_ response speed was improved greatly compared with previously reported results. Therefore, due to its strong sensitivity, nontoxicity, low price, and biodegradability, the prepared pH-sensitive film can be utilized in the real-time monitoring of meat freshness, and it has great potential as an intelligent packaging material for monitoring food hygiene and safety.

## Figures and Tables

**Table 1:** Mechanical performance and barrier properties of pH-sensitive films.

Film	Thickness (mm)	Mechanical Properties		Barrier Properties	
		TS (MPa)	EB (%)	WVP (g/m^2^·24 h)	OP (cm^3^/m^2^·24 h·0.1 MPa)
ASKG/SPI	0.056 ± 0.001 ^d^	1.543 ± 0.120 ^d^	202.292 ± 2.567 ^a^	560.22 ± 0.04 ^c^	1.024 ± 0.013 ^b^
LRMA-1%	0.081 ± 0.008 ^c^	4.874 ± 0.259 ^b^	12.395 ± 2.394 ^d^	746.41 ± 0.02 ^b^	0.156 ± 0.025 ^c^
LRMA-2%	0.092 ± 0.003 ^bc^	3.505 ± 0.122 ^c^	25.054 ± 2.843 ^c^	915.19 ± 0.01 ^a^	0.160 ± 0.032 ^c^
LRMA-3%	0.118 ± 0.001 ^a^	3.863 ± 0.476 ^c^	86.150 ± 2.609 ^b^	450.30 ± 0.01 ^d^	4.755 ± 0.017 ^a^
LRMA-4%	0.098 ± 0.010 ^ab^	8.147 ± 1.764 ^a^	5.560 ± 2.833 ^e^	446.36 ± 0.02 ^d^	4.716 ± 0.036 ^a^

Note: All data are the mean ± standard deviation; ^a–e^ different superscript letters in the same parameters indicate significant differences (*p* < 0.05).

**Table 2:** The colorimetric parameters and photographs of pH-sensitive films in different buffer solutions (2–10).

Sample	pH	L*	a*	b*	Photographs	
					Before Sensing	After Sensing
LRMA-1%	2 3 4 5 6 7 8 9 10	86.31 ± 1.98 ^c^ 76.13 ± 2.14 ^a^ 82.72 ± 3.12 ^bc^ 88.09 ± 2.66 ^c^ 96.73 ± 4.95 ^d^ 77.34 ± 4.63 ^ab^ 83.58 ± 1.08 ^c^ 86.58 ± 2.67 ^c^ 94.85 ± 1.95 ^d^	11.86 ± 1.00 ^f^ 22.94 ± 0.96 ^g^ 8.38 ± 0.38 ^e^ 7.55 ± 0.25 ^e^ 0.78 ± 0.03 ^a^ 7.55 ± 0.51 ^e^ 4.85 ± 0.10 ^c^ 6.12 ± 0.27 ^d^ 2.17 ± 0.21 ^b^	1.48 ± 0.14 ^c^ −5.31 ± 0.25 ^a^ 3.12 ± 0.03 ^e^ 2.49 ± 0.15 ^d^ 1.40 ± 0.04 ^c^ 2.49 ± 0.18 ^d^ 3.58 ± 0.22 ^f^ 5.68 ± 0.27 ^g^ 0.06 ± 0.01 ^b^	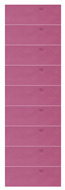	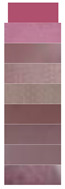
LRMA-2%	2 3 4 5 6 7 8 9 10	68.66 ± 1.22 ^c^ 62.08 ± 1.37 ^b^ 79.57 ± 1.37 ^d^ 69.81 ± 3.13 ^c^ 89.42 ± 0.45 ^g^ 59.67 ± 1.08 ^a^ 61.56 ± 0.35 ^b^ 73.94 ± 2.11 ^e^ 83.47 ± 0.22 ^f^	33.09 ± 0.42 ^g^ 37.30 ± 0.11 ^h^ 11.73 ± 0.31 ^d^ 16.58 ± 076 ^d^ 3.69 ± 0.03 ^a^ 17.16 ± 1.09 ^ef^ 18.53 ± 0.72 ^f^ 8.67 ± 0.14 ^c^ 4.61 ± 0.16 ^b^	−6.47 ± 0.49 ^b^ −12.39 ± 0.30 ^a^ 5.42 ± 0.11 ^de^ 6.60 ± 0.36 ^e^ 0.32 ± 0.09 ^c^ −11.47 ± 1.13 ^a^ −13.03 ± 0.81 ^a^ 3.71 ± 0.55 ^d^ −0.10 ± 0.07 ^c^	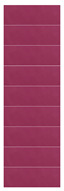	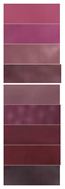
LRMA-3%	2 3 4 5 6 7 8 9 10	57.98 ± 0.69 ^d^ 54.96 ± 1.38 ^c^ 69.61 ± 1.62 ^e^ 75.50 ± 1.96 ^f^ 74.02 ± 2.20 ^f^ 35.04 ± 0.93 ^a^ 48.37 ± 0.76 ^b^ 58.15 ± 1.05 ^d^ 55.65 ± 1.28 ^c^	56.66 ± 1.77 ^h^ 37.34 ± 1.12 ^e^ 19.8 ± 0.08 ^b^ 19.28 ± 1.73 ^b^ 9.99 ± 1.01 ^a^ 22.27 ± 0.35 ^c^ 46.93 ± 0.99 ^f^ 28.69 ± 0.56 ^d^ 51.57 ± 1.57 ^g^	−13.92 ± 0.38 ^a^ −9.01 ± 1.85 ^b^ 2.56 ± 1.25 ^d^ 1.00 ± 0.25 ^d^ 2.53 ± 0.28 ^d^ −7.54 ± 1.13 ^b^ −13.42 ± 0.74 ^a^ −4.25 ± 0.66 ^c^ −14.42 ± 1.07 ^a^	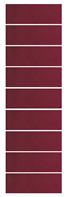	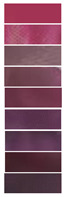
LRMA-4%	2 3 4 5 6 7 8 9 10	45.00 ± 1.95 ^b^ 47.10 ± 1.51 ^cd^ 62.24 ± 1.98 ^e^ 40.55 ± 1.26 ^a^ 45.51 ± 0.76 ^bc^ 47.49 ± 0.45 ^d^ 44.78 ± 1.35 ^b^ 63.32 ± 0.78 ^e^ 47.26 ± 1.29 ^cd^	65.24 ± 0.22 ^g^ 49.23 ± 0.36 ^d^ 24.83 ± 0.59 ^a^ 52.45 ± 0.36 ^e^ 55.45 ± 0.17 ^f^ 34.93 ± 1.14 ^b^ 50.27 ± 1.28 ^d^ 43.37 ± 0.46 ^c^ 53.67 ± 0.52 ^e^	−4.05 ± 1.01 ^d^ −2.31 ± 1.02 ^e^ 0.80 ± 0.09 ^f^ −15.50 ± 2.35 ^a^ −13.45 ± 0.25 ^b^ −10.96 ± 0.06 ^c^ −11.03 ± 0.75 ^c^ −12.16 ± 0.88 ^bc^ −13.72 ± 0.73 ^b^	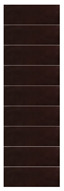	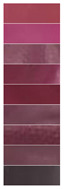

Note: All data are the mean ± standard deviation; ^a–h^ different superscript letters in the same parameters indicate significant differences (*p* < 0.05).

## Data Availability

The data that support the findings of this study are available from the corresponding author upon reasonable request.
